# Research on Dry Coupling Technology in the Ultrasonic Non-Destructive Testing of Concrete

**DOI:** 10.3390/mi16010072

**Published:** 2025-01-10

**Authors:** Jun Li, Zeyu Chen

**Affiliations:** 1 College of Mechanical & Electrical Engineering, Central South University, Changsha 410083, China; lijun202501@163.com; 2 China Railway 11th Bureau Group Co., Ltd., Wuhan 272199, China

**Keywords:** ultrasonic transducer, non-destructive testing, ultrasonic imaging

## Abstract

In the health monitoring and safety assessments of concrete structures, ultrasonic non-destructive testing (NDT) technology has become an indispensable tool due to its non-destructive nature, efficiency, and precision. However, when used in inspecting irregular concrete surfaces, traditional planar ultrasonic transducers often encounter energy loss and signal attenuation induced by poor interface coupling, which significantly reduces the accuracy and reliability of the test results. To address this problem, this article proposes a point-contact dry coupling ultrasonic transducer solution, which enables efficient acquisition of ultrasonic signals within concrete without the need for couplants. By combining an array imaging system with a total focusing algorithm, this study not only significantly enhances the convenience and signal-to-noise ratio (SNR) of concrete ultrasonic imaging, but also opens new pathways for ultrasonic NDT technology in concrete.

## 1. Introduction

Concrete serves as the cornerstone of the construction industry. The health of its internal structure is directly related to the safety and stability of buildings [1,2]. Therefore, it is particularly important to accurately detect internal defects in concrete. Since Soviet scholar S.Y. Sokolov first proposed using ultrasonic waves to detect internal defects in opaque materials in 1929 [3], ultrasonic non-destructive testing (NDT) technology has gained significant attention for its unique advantages in concrete testing [4,5]. However, the application of traditional planar ultrasonic transducers still faces numerous challenges in the case of uneven concrete surfaces [6,7,8,9]. This study aims to overcome these challenges and improve the efficiency and accuracy of ultrasonic testing of concrete by introducing point-contact dry coupling ultrasonic transducer technology.

Frederick et al. [10] first reported a computer-controlled synthetic aperture focusing imaging device in 1976, showcasing the enormous potential of synthetic aperture focusing technology (SAFT) in enhancing azimuthal resolution for long-distance target detection. In 1995, Schickert [11] successfully applied ultrasonic SAFT to the two-dimensional cross-sectional imaging of concrete components. This application effectively enabled better visualization of concrete’s internal structure, providing an important reference for subsequent research. Silverstein and Tomas [12] introduced sensor signal processing method into synthetic aperture ultrasonic imaging in 1993, which greatly enhanced imaging clarity and accuracy. In 2007, Tong et al. [13] used transient elastic waves generated using steel ball impacts to achieve greater detection depths in SAFT imaging of concrete structures, thereby improving the capability of ultrasound in detecting complex concrete structures. However, due to the complex internal structure of concrete, distinguishing effective signals from stray ones remains a challenge that limits the application of ultrasound in concrete NDT.

Ribay et al. [14] experimentally detected concrete and reinforced concrete models using an array probe composed of 4 × 4 transducer elements, validating its effectiveness in detecting complex concrete structures. And Krause et al. [15] designed an ultrasonic probe array consisting of ten transducers, each with a diameter of 50 mm, and stuck it to the concrete surface with a certain template device for detection. This ultrasonic probe array demonstrated exceptional efficiency, with data acquisition at each detection point taking only 3 min, thus enabling a comprehensive scan of one square meter of concrete within approximately 2 h. Additionally, the detection frequency of the probe ranges from 50 kHz to 250 kHz, fully meeting various detection needs. Compared to a single probe, transducer array technology exhibits excellent performance in enhancing the signal-to-noise ratio (SNR) of system imaging through the collaborative work of multiple probes. Innovations in array technology have not only improved the accuracy and efficiency of concrete detection, but have also opened new research directions and application prospects in NDT.

However, when dealing with uneven concrete surfaces, traditional planar ultrasonic transducers face energy loss at the interface due to the poor fit between the transducer surface and the concrete surface. Additionally, during the propagation of ultrasonic waves within concrete, complex mode conversions occur, leading to enhanced structural noise and complicated boundary reflections. These factors collectively cause a significant reduction in the SNR of traditional ultrasonic testing technology. The issues of low energy transfer efficiency and significant signal attenuation directly undermine the accuracy and reliability of the testing results, posing challenges for the precise identification of concrete’s internal defects.

To address the aforementioned issues, researchers proposed the point-contact dry coupling ultrasonic transducer solution. By employing a point-contact method, this solution effectively avoids the interface coupling problems that traditional planar transducers face on uneven surfaces, thereby reducing energy loss and signal attenuation. Commercial transducers typically operate at a frequency of 50 kHz. However, there are a lack of analyses of their frequency responses. In this study, the vibration mode of an ultrasonic transducer was established, and its frequency response was analyzed. An ultrasonic transducer was designed and manufactured with a resonant frequency of 110 kHz.

In addition, by adopting a total focusing imaging algorithm that integrates variational mode decomposition (VMD) and wavelet packet transform (WPT), this approach achieves precise control and efficient processing of ultrasonic signals, further improving the resolution of ultrasonic imaging in concrete.

## 2. Simulation and Experiment

The research utilizes COMSOL Multiphysics (version 6.0, COMSOL AB, Stockholm, Sweden) for the modeling and simulation of ultrasonic transducers. The geometric model of the ultrasonic transducer’s structure is imported into COMSOL’s geometry tools, ensuring that the model’s dimensions and shape align with the actual device. Subsequently, material properties such as sound velocity, density, and Young’s modulus are defined in the material module, and the boundary conditions are applied.

Once these preparations are complete, the eigenfrequency, frequency response, and displacement of the transducer can be solved. The impedance–phase curve of the transducer can also be generated within the specified frequency range.

In the frequency domain simulation, this study systematically investigated the propagation behavior of ultrasonic waves within the concrete medium. The focus was on analyzing the attenuation patterns, energy distribution, and scattering effects of ultrasonic waves at different frequencies. Through detailed simulation analyses and comparisons, the optimal operating frequency range for this research was determined, providing a solid theoretical foundation for the subsequent experimental design and implementation.

**Figure 1:**
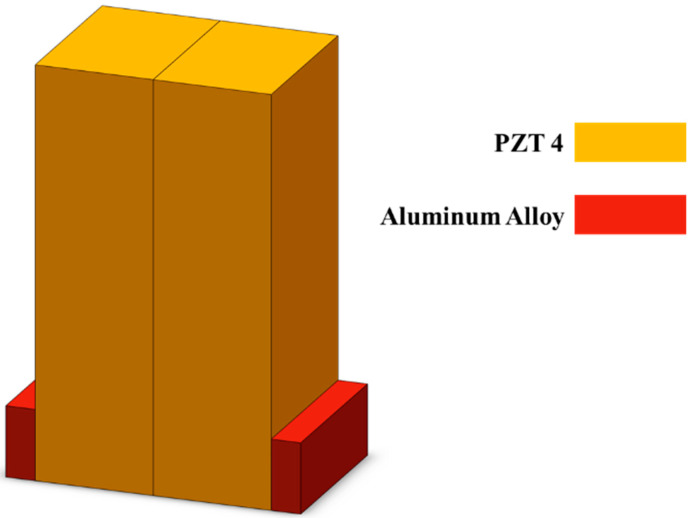
Schematic diagram showing the structural simulation of the ultrasonic transducer.

**Figure 2:**
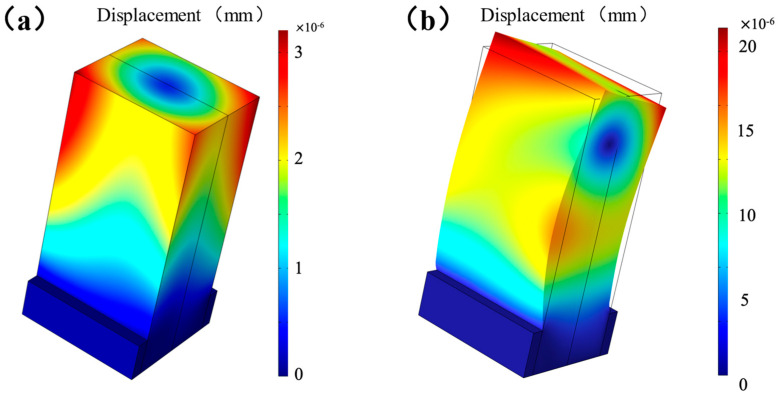
Vibration mode of the transducer for concrete at (**a**) 50 kHz and (**b**) 110 kHz.

**Figure 3:**
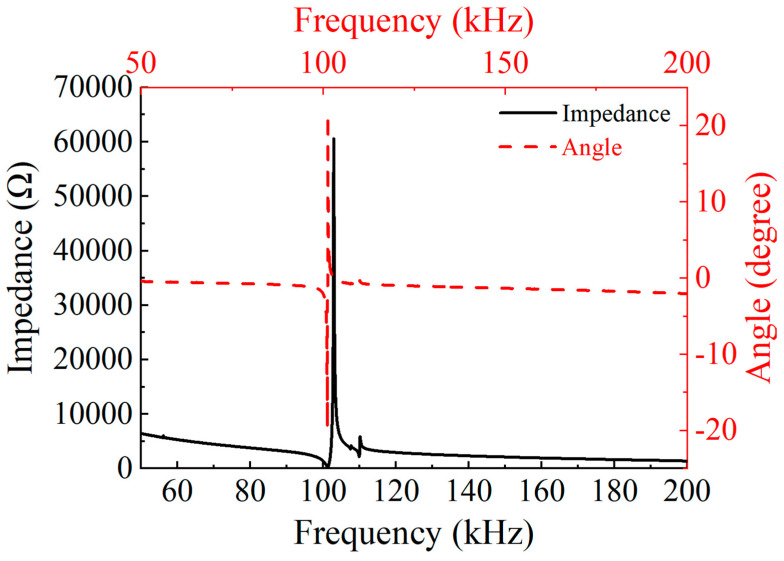
Impedance spectrum of concrete sensor obtained using the simulation.

As shown in Figure 1, the ultrasonic transducer is simplified as a composite structure consisting of piezoelectric material and structural steel blocks. A vibration mode analysis was performed for a shear wave ultrasonic transducer. During the analysis, the metal-piezoelectric material contact interface was specially set as fixed boundary conditions, while other parts were set as free conditions. Table 1 lists the specific simulation parameters for the piezoelectric material and structural steel in the model, where the aluminum alloy mainly serves to fix the positions of the two piezoelectric ceramics. As illustrated in Figure 2, the vibration mode analysis model of the piezoelectric material clearly displays its torsional vibration mode. The simulation results in Figure 3 show that the first resonance frequency of the ultrasonic converter was 101.3 kHz and the second resonance frequency was 109.9 kHz.

**Figure 4:**
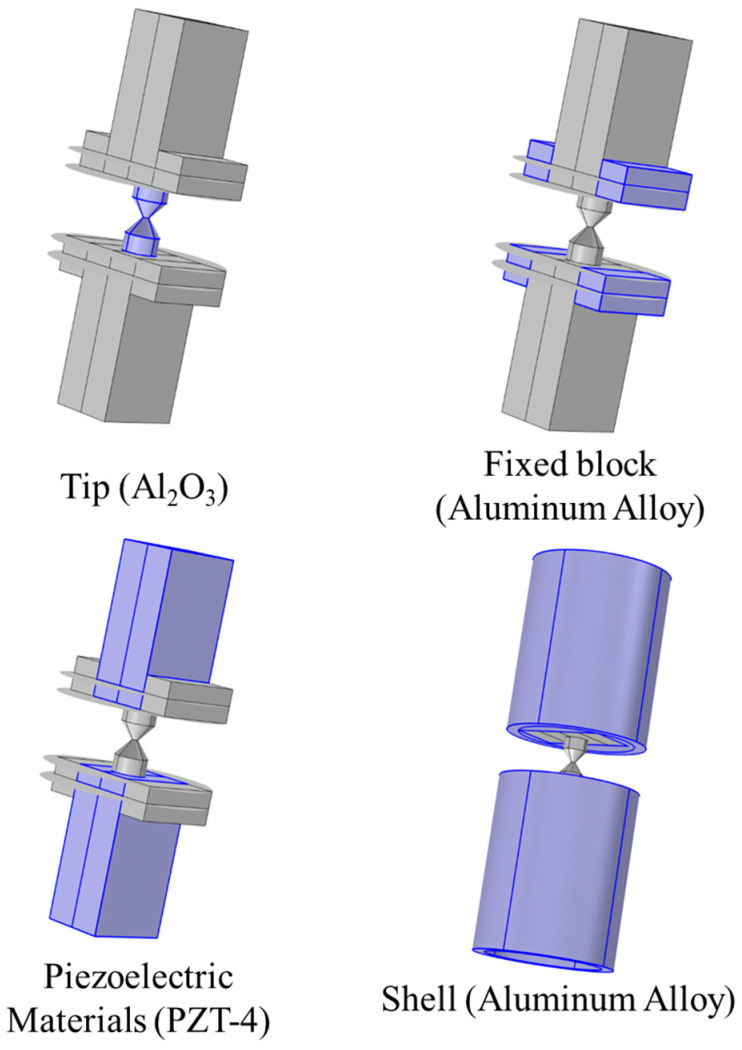
Schematic diagram showing the time-domain simulation model.

The material parameters of each component in the time-domain simulation model depicted in Figure 4 are detailed in Table 1 The core structure of the model consists of four parts: the tip, the fixed block, the piezoelectric materials, and the shell. To simulate the transmitting and receiving process of ultrasonic waves, two simulation devices are connected via their tips. One device acts as the ultrasonic wave transmitter excited by a single-cycle sinusoidal wave signal with a frequency of 100 kHz; the other serves as the receiver, which receives the ultrasonic wave signal from the transmitter.

**Figure 5:**
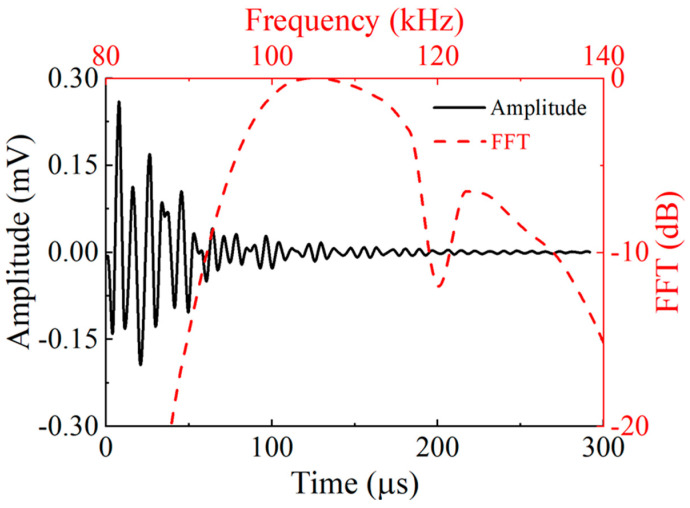
The waveform received using the receiving transducer.

After performing a spectral analysis of the waveform received by the receiving transducer, it was found that the frequency of the received echo signal is approximately 110 kHz, which is shown in Figure 5.

**Figure 6:**
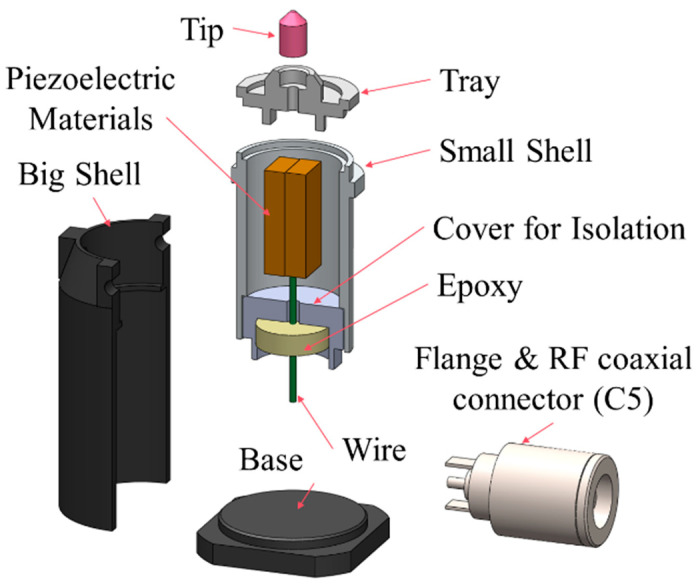
Structure of the ultrasonic transducer.

Figure 6 illustrates the detailed structure of the shear wave ultrasonic transducer. This transducer integrates several key components to achieve its function. Specifically, it mainly includes the following parts:

- Tip: As the connection point for ultrasonic wave transmission, it is responsible for efficiently transferring ultrasonic vibrations from the transducer into the concrete medium under test.
- Tray: It provides stable support not only for fixing the contact tip, but also for firmly connecting the piezoelectric material and providing a connection point with the small shell.
- Piezoelectric material: As the core component of the transducer, it efficiently converts electrical signals into mechanical vibrations to generate the required ultrasonic vibrations.
- Small shell: It is designed to secure the tray, offer a protective space for the piezoelectric material, and effectively shield against external noise interference to ensure signal purity.
- Isolation cover: The isolation cover is used to secure the cable and isolate the area between the small shell and the big shell. Meanwhile, it further enhances the structure’s sealing integrity and stability.
- Big shell: It houses the connector and other electronic components, providing a robust outer casing for the overall structure while ensuring compatibility with the testing fixture.
- RF coaxial connector (C5): It serves as a standard interface for easy connection with external devices, facilitating signal input and output.
- Flange: It forms a stable physical connection between the socket and the large shell, enhancing the overall structural stability.
- Cover and spring: The cover directly contacts the spring. During testing, the spring’s cushioning action ensures uniform contact between the ultrasonic transducer and the surface of the concrete being tested, improving the accuracy and reliability of the measurement.

The preparation process for the ultrasonic transducer is as follows. First, use a quasi-static d_33_ meter (Shanghai Yidian (Group) Co., Ltd., Shanghai, China) to accurately measure the polarity of the piezoelectric material and mark it clearly. After that, select high-temperature conductive adhesive Loctite 3880 (Henkel, Düsseldorf, Germany) to bond electrodes’ surfaces with the same polarity. Heat the adhesive with a hot air gun at 125 °C for 15 min to ensure a strong bond, and connect the electrodes with lead wires. For the opposite polarity side of the piezoelectric material, connect two pieces of material in series with lead wires and extend a wire for subsequent connection. Apply 301 epoxy resin to cover and protect the exposed parts of the piezoelectric material connection cable. Precisely bond the piezoelectric material to the center of the tray, ensuring stable positioning. Then, securely attach the tray to the small shell to form an initial protective structure. After that, carefully place the isolation cover into the small shell, ensuring that the cable can pass through the pre-cut holes in the isolation cover. Use 301 epoxy resin to fill the gap between the isolation cover and the small shell to enhance overall sealing integrity and stability. Next, properly position the C5 socket and the internal arc flange inside the large shell, and solder the cable to the C5 socket to establish electrical connection. Then, fix the internal and external arc flanges with epoxy resin to ensure a compact and durable structure. Also, apply epoxy resin evenly to the contact surfaces between the large shell and the cover. Finally, place the entire assembly in an oven and cure it at 60 °C for two hours to ensure all components are tightly bonded. The ultrasonic transducer is then fabricated completely.

**Figure 7:**
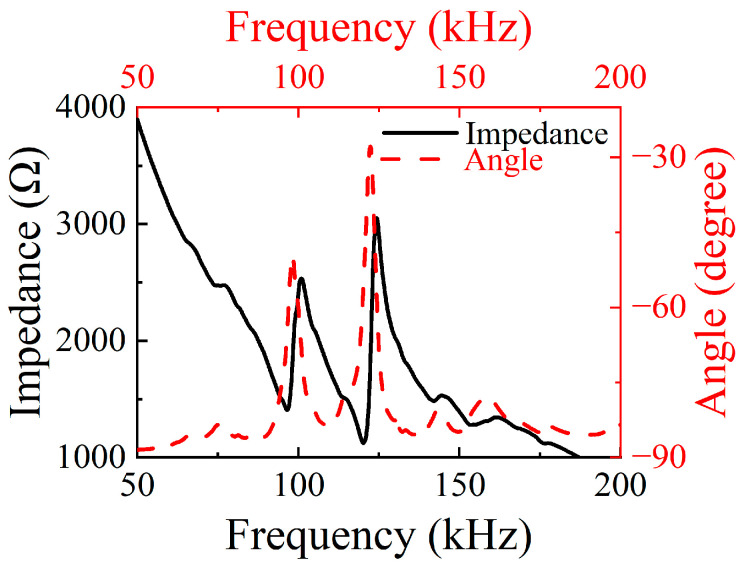
Impedance spectrum of the ultrasonic transducer.

During the electroacoustic testing of the ultrasonic transducer, the impedance characteristics of the transducer were measured in detail using an impedance analyzer. The ultrasonic transducer was connected to the impedance analyzer, and the testing range was set to 50–200 kHz. The impedance spectrum of the transducer is shown in Figure 7, from which it can be seen that the first resonance frequency of the ultrasonic converter is 96.6 kHz, with the impedance value at this frequency being 1405 Ω, and the second resonance frequency is 120.3 kHz, with the impedance value at this frequency being 1125 Ω.

**Figure 8:**
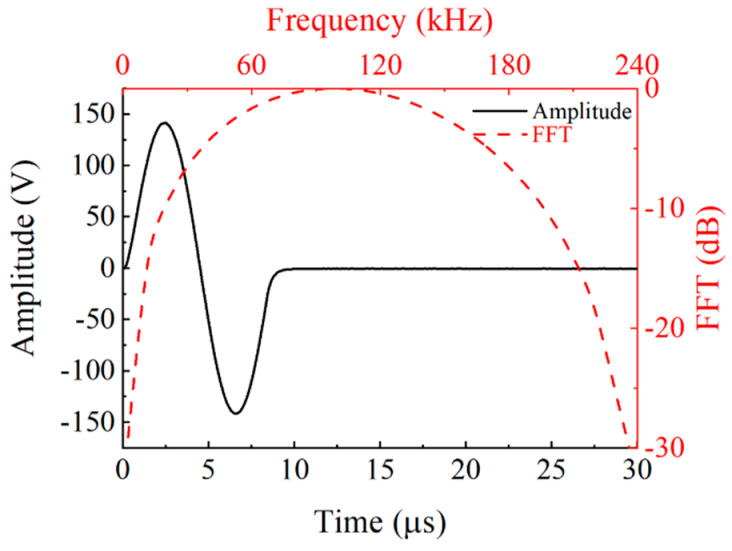
Excitation waveform and spectrum of the ultrasonic transducer.

**Figure 9:**
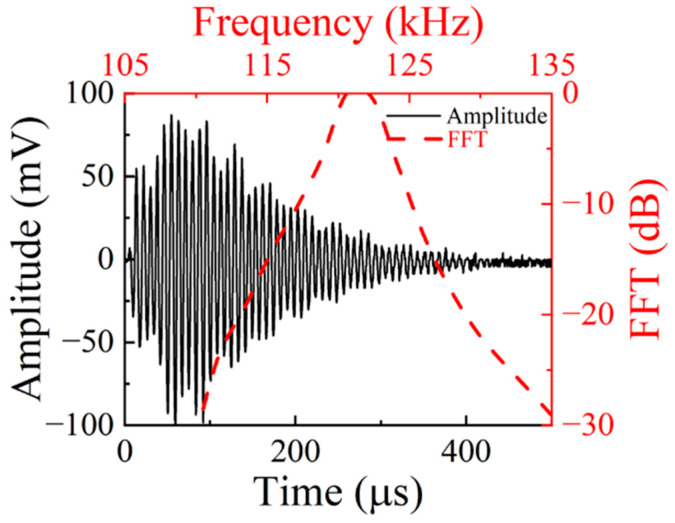
Echo signal received using the ultrasonic transducer.

Two ultrasonic transducers’ tips were connected. A signal generator AFG 1022 (Tektronix, Beaverton, OR, USA) was used to produce a single-cycle sinusoidal wave signal at a frequency of 120 kHz. This signal was amplified using a power amplifier ATA-2041 (Xi’an Aigtek Electronic Co., Ltd., Xi’an, China) to a peak value of 285 V to excite the prepared ultrasonic transducer. The received signal was collected using a multi-channel recorder MR6000 (HIOKI, Corporation, Nagano, Japan). Figure 8 illustrates the excitation waveform, and Figure 9 shows the waveform of the echo signal received by the transducer.

**Figure 10:**
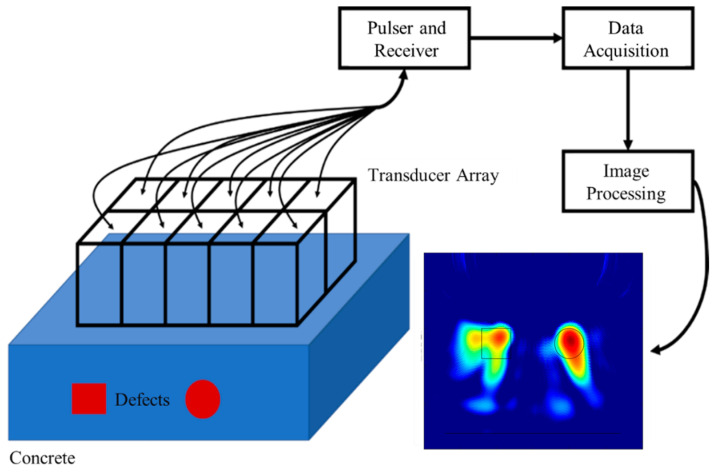
Schematic diagram showing the structure of the ultrasonic imaging system for concrete.

**Figure 11:**
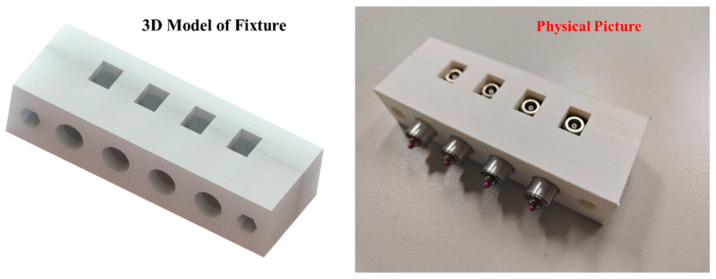
Ultrasonic array fixture.

As shown in Figure 10, the ultrasonic transducer array is the core component of the ultrasonic imaging system for concrete. It works closely with the pulse transmit–receive module to perform the transmission and reception of ultrasonic waves. As shown in Figure 11, the element spacing within the ultrasonic array is 2 cm, and a spring is used between the elements and the fixture to ensure tight contact between the transducer and the concrete.

The pulse transmit–receive module is divided into two main submodules: the pulse transmit module, and the pulse receive module. The transmit module sends high-voltage pulse signals to the specified elements in the ultrasonic transducer array. These pulse signals are converted by the transducer into ultrasonic waves, which then penetrate the concrete medium. When the ultrasonic waves encounter obstacles within the concrete (such as voids, cracks, or rebar), they are reflected. These reflected ultrasonic wave signals are then captured by the elements in the array and converted into electrical signals. The acquisition module digitizes the echo signals collected by each element, converting them into digital signals that can be further analyzed. The digital signals are subsequently transmitted to an image processing and display module for back-end processing.

**Figure 12:**
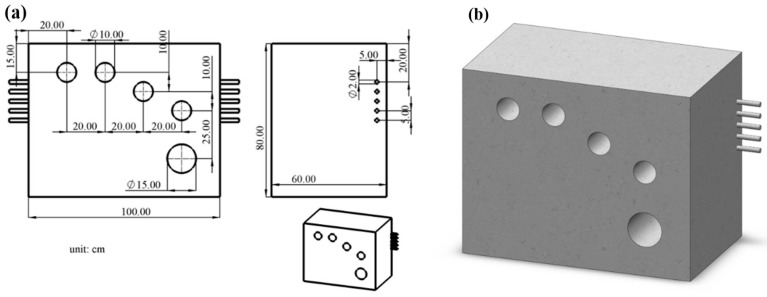
(**a**) Engineering drawing of the concrete test block. (**b**) Model diagram of the concrete test block.

To comprehensively evaluate the performance of the ultrasonic array in concrete imaging, we designed a C15 concrete test block that is 100 cm long, 60 cm wide, and 80 cm high, with various simulated defects. The front face of the test block features four cavities, each with a diameter of 10 cm, arranged sequentially from left to right, as well as one larger cavity with a diameter of 15 cm, to simulate different types of air defects. Additionally, the side of the test block is equipped with five steel bars, each with a diameter of 2 cm, evenly spaced at 5 cm intervals and located 5 cm from the surface of the test block, to simulate metal structure defects within the concrete. A detailed layout of the test block is illustrated in Figure 12a, and the concrete model is shown in Figure 12b.

**Figure 13:**
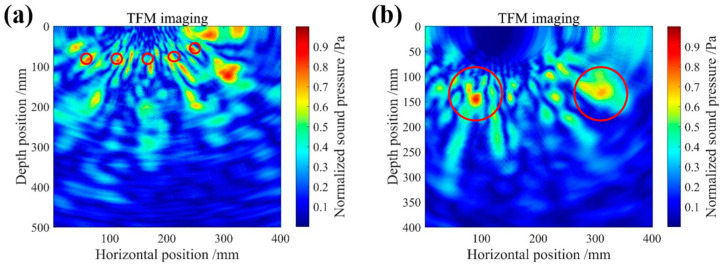
(**a**) Steel bar defect TFM imaging. (**b**) Air void TFM imaging.

**Figure 14:**
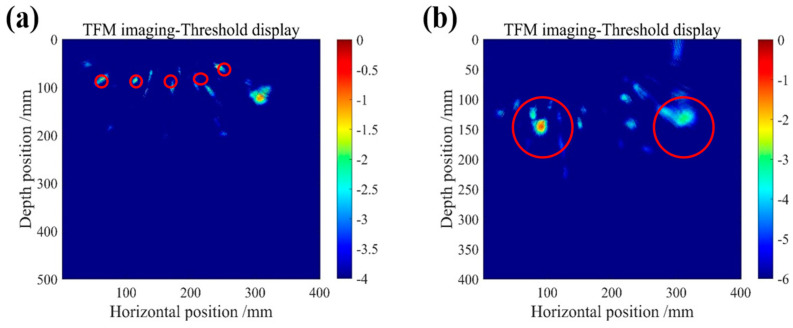
(**a**) Threshold display under steel bar defect TFM imaging. (**b**) Threshold display under air void TFM imaging.

The imaging test utilized an array system comprising eight ultrasonic transducers. Each time, one transducer was activated as the transmitter while the remaining seven served as receivers. To enhance signal quality, we innovatively combined VMD and WPT, effectively reducing noise on the received echo signals. The total focusing method (TFM) was used for high-precision imaging. The internal structural features of the test block were clearly shown in the imaging results. Figure 13a precisely captures the fine steel bar contours distributed along the side surface of the test block, while Figure 13b accurately presents the two prominent cavity defects on the left side of the test block’s front face (the defects are marked with red lines). To further highlight the locations of minor defects, image threshold adjustments were made, resulting in more intuitive imaging results. As shown in Figure 14, both air voids and steel bar details are clearly displayed.

The imaging results demonstrate that the ultrasonic transducer designed and prepared in this study exhibited outstanding performance; it successfully identified and accurately located air voids and steel bar defects within the concrete test block. By adjusting the display threshold of the imaging results, the transducer can not only clearly reveal air voids with diameters ranging from 10 cm to 15 cm, but also precisely identify steel bars with a diameter of 2 cm.

## 3. Conclusions

The proposed point-contact dry-coupling ultrasonic transducer solution and array imaging system have shown significant advantages in ultrasonic NDT of concrete. This approach not only addresses the challenges faced by traditional planar transducers on uneven surfaces, but also enhances the accuracy and reliability of the testing results through the TFM imaging algorithm. This study provides new technological methods and insights for concrete NDT, offering both theoretical value and practical significance. Future research may further explore the potential applications of this technology in complex concrete structures and its integration with other NDT techniques.

## Figures and Tables

**Table 1:** Simulation parameters of the dry coupling ultrasonic transducer for concrete.

Material	PZT-4	Aluminum Alloy
Density	7500 kg/m^3^	7850 kg/m^3^
Young’s Modulus	1.15 × 10^11^ Pa	2 × 10^11^ Pa
Poisson’s Ratio	-	0.3
Dimension	9.2 × 5 × 2 mm^3^	5 × 1.5 × 0.5 mm^3^

## Data Availability

The original contributions presented in this study are included in the article. Further inquiries can be directed to the corresponding author.
